# Application of Nanomaterials in Efficient Energy Conversion and Storage

**DOI:** 10.3390/nano15211635

**Published:** 2025-10-27

**Authors:** Gege He

**Affiliations:** State Key Laboratory of Chemistry and Utilization of Carbon Based Energy Resources, School of Chemical Engineering and Technology, Xinjiang University, Urumqi 830017, China; gghe01@xju.edu.cn

## 1. Introduction

The global energy landscape is undergoing a profound transformation driven by the urgent need to transition from fossil fuels to sustainable and renewable energy sources [1]. This shift is critical for addressing climate change, reducing carbon emissions, and achieving the ambitious “dual carbon” goals of carbon peak and carbon neutrality. At the heart of this energy revolution lie advanced nanomaterials, which have emerged as enablers for next-generation energy conversion and storage technologies [2]. Nanostructured materials offer unique advantages including high surface-to-volume ratios, tunable transport properties, altered physical characteristics, and quantum confinement effects that are particularly beneficial for energy applications [3]. The integration of nanotechnology in energy systems has led to significant improvements in the performance of solar cells, catalysts, thermoelectrics, lithium-ion batteries, supercapacitors, and hydrogen storage systems [4]. These advancements are achieved through various mechanisms: providing large surface areas to boost electrochemical reactions or molecular adsorption, generating enhanced optical effects to improve light absorption, and creating highly crystalline and porous structures to facilitate electron/ion transport and electrolyte diffusion [5]. Recent research has focused on optimizing these nanomaterials for greater efficiency, sustainability, and cost-effectiveness, pushing the boundaries of what is possible in energy conversion and storage.

The growing interest in this field is reflected by the numerous scientific publications and Special Issues dedicated to nanomaterials for energy applications. This Editorial aims to provide an overview of recent advances in this rapidly evolving field, highlighting representative studies that demonstrate the diverse applications of nanomaterials in addressing our global energy challenges.

## 2. An Overview of the Published Articles

The current research landscape in nanomaterials for energy applications reveals significant advances across multiple domains, from solar energy harvesting to electrochemical energy storage and beyond. The selected publications in this Special Issue reflect the diversity and innovation characterizing this field.

Yu Lu et al. [6] developed a Pt single-atom catalyst (SAC) on a MOF-derived carbon substrate using a two-step laser annealing process. With an ultralow Pt loading of 0.86 wt%, the catalyst demonstrated exceptional performance for the hydrogen evolution reaction (HER) in acid, exhibiting a mass activity 20.52 times higher than that of commercial Pt/C at an overpotential of 50 mV. The synthesis involves sequential IR and UV laser irradiation to pyrolyze precursors and achieve atomic dispersion of Pt. This method is presented as a simple, fast, and potentially scalable approach for mass-producing high-performance SACs [6]. Chenxing Wang et al. [7] investigate the synthesis of electrochemical titanium oxide nanostructures via three anodization methods—nanodot, lateral, and imprint—for neuromorphic applications. Mathematical modeling revealed dynamic conductivity channel formation and evolution during growth, including channel breakup and nucleation events specific to each method. XPS analysis confirmed the predicted gradient oxide composition, with TiO_2_ dominating near the surface and lower oxides increasing with depth. All nanostructures exhibited stable resistive switching over 1000 cycles and retained their state for 10,000 s, demonstrating their potential for neuromorphic devices [7]. Chan Ju Choi et al. [8] demonstrate that incorporating well-dispersed multi-walled carbon nanotubes (MWCNTs) into high-nickel cathodes significantly enhances the performance of lithium-ion batteries. Using ultrasonication for uniform dispersion, MWCNTs formed an efficient conductive network, improving both electronic and ionic conductivity while reducing internal resistance. Electrodes with 2 wt% MWCNTs exhibited superior rate capability at high current density and achieved 89.5% capacity retention over cycling. The findings highlight MWCNTs as an effective conductive additive for developing high-energy-density electrodes with improved stability and kinetics [8]. Md Yusuf Ali et al. [9] present the synthesis of Y-doped NASICON-type LATP solid electrolytes via spray-flame synthesis, yielding nanoparticles that crystallize into the desired phase after brief annealing. Yttrium doping was found to increase the crystal volume and significantly enhance ionic conductivity, reaching 0.84 mS/cm at room temperature—over eight times higher than the undoped sample. Although secondary phases such as YPO_4_ and LiTiOPO_4_ formed at higher doping levels and temperatures, the materials exhibit promising properties for solid-state batteries, particularly due to their high ionic conductivity and low required sintering temperature [9]. Irene Lau et al. [10] demonstrate a method for fabricating planar, interdigitated Li-S batteries using laser-induced graphene (LIG) as both the cathode and anode scaffold. Sulfur was deposited on the cathode fingers via selective nucleation and melt imbibition, while lithium was electrodeposited on the anode fingers using a silver-seeded pulse-reverse-pulse technique, achieving high loadings without short-circuiting. The resulting flexible, binder-free full cell delivered a capacity of over 1 mAh/cm^2^ and an energy density of 200 mWh/cm^3^. Although cycling stability was limited by lithium degradation, the interdigitated LIG architecture provides a valuable platform for fundamental studies of Li-S and related battery systems [10].

## 3. Conclusions

Among the main goals of the authors of this Special Issue, the development of more efficient and sustainable energy materials is highlighted. The contributions demonstrate that innovative nanostructure design and advanced synthesis methods—such as laser annealing, anodization patterning, ultrasonically assisted dispersion, spray-flame synthesis, and laser-induced graphene processing—can significantly improve electrochemical performance while reducing or replacing critical raw materials. These advances lead to higher catalytic activity, enhanced ionic conductivity, greater cycling stability, and new architectural possibilities across systems including fuel cells, batteries, and neuromorphic devices. Nevertheless, challenges such as material degradation and component stability under operational conditions remain, underscoring the need for continued research into durable and scalable nanomaterial designs. Ultimately, technological progress in sustainable energy applications remains closely linked to breakthroughs in nanomaterial science and engineering.
